# CD10 expression in urothelial carcinoma of the bladder

**DOI:** 10.1186/1746-1596-4-38

**Published:** 2009-11-16

**Authors:** Burak Bahadir, Kemal Behzatoglu, Sibel Bektas, Erol R Bozkurt, Sukru O Ozdamar

**Affiliations:** 1 Department of Pathology, Zonguldak Karaelmas University, Faculty of Medicine, Zonguldak, Turkey; 2 Department of Pathology, Istanbul Educational Hospital, Istanbul, Turkey

## Abstract

**Background:**

CD10 antigen is a 100-kDa-cell surface zinc metalloendopeptidase and it is expressed in a variety of normal and neoplastic lymphoid and nonlymphoid tissues. The aim of this study was to evaluate CD10 expression in urothelial carcinoma of the urinary bladder and to determine the correlation between immunohistochemical (IHC) CD10 expression and histopathologic parameters including grade and stage.

**Methods:**

371 cases of urothelial bladder carcinomas, all from transurethral resections, were included in this study. Hematoxylin-eosin (HE) stained sections from each case were reevaluated histopathologically according to WHO 2004 grading system. The TNM system was used for pathologic staging. Selected slides were also studied by IHC and a semiquantitative scoring for CD10 expression based on the percentage of positive cells was performed.

**Results:**

157 cases (42.3%) showed immunostaining while 214 cases (57.7%) were negative for CD10. 1+ staining was seen in 65 CD10 positive cases (41.4%), and 2+ in 92 cases (58.6%). Overall CD10 expression as well as 2+ immunostaining was significantly correlated with high histologic grade. Overall CD10 expression was also significantly higher in invasive pT1 and pT2-3 tumors compared to noninvasive pTa tumors. pT1 and pT2-3 tumors were also significantly correlated with 2+ immunostaining.

**Conclusion:**

To date, only a few comparative IHC studies have assessed CD10 expression in urothelial carcinoma of the urinary bladder and this study represents the largest series. Our findings indicate that CD10 expression is strongly correlated with high tumor grade and stage in urothelial carcinoma of the bladder, and that CD10 may be associated with tumor progression in bladder cancer pathogenesis.

## Background

Cancer of the bladder represents the ninth most common cause of cancer worldwide and the 13^th ^most numerous cause of death from cancer with an estimated 357,000 newly diagnosed cases and 145,000 cancer-related deaths in 2002 [1]. Most patients (70-80%) with newly diagnosed urothelial carcinoma of the bladder present with well-differentiated superficial papillary tumors. Prolonged survival in most patients with superficial cancers is achieved by transurethral resection (TUR) with or without intravesical chemotherapy [2-6]. Nonetheless, these patients still have a high risk of recurrence following initial resection [7]. The major prognostic factors in carcinoma of the bladder are the depth of invasion into the bladder wall and the degree of differentiation of the tumor. However, there is no reliable parameter predicting the risk of recurrence or progression. Molecular markers are, therefore, required to estimate the individual prognosis of patients as well as for effective diagnosis and treatment.

CD10 is a single-chain, 90-110-kDa cell surface zinc-dependent metalloprotease that inactivates various bioactive neuropeptides. Terms synonymous with CD10 include common acute lymphocytic leukemia antigen (CALLA), enkephalinase, membrane metalloendopeptidase (MME), membrane-associated neutral endopeptidase (NEP 24.11), and neprilysin [8-10]. Initially it was discovered on the surface of acute lymphoblastic leukemia cells, and considered to be a tumor-specific antigen [11]. It was then found that a variety of normal cells including cells of nonhematopoietic origin such as glomerular and proximal tubular epithelium of the kidney, liver, breast myoepithelium, and lung, as well as fibroblasts and cells of the central nervous system are able to express CD10 [8,9,12-18]. CD10 is expressed by a number of hematopoietic cells such as immature T and B cells, B cells of the germinal centers of lymphoid follicles, and granulocytes as well as lymphoid malignancies including the majority of acute lymphoblastic leukemia, and also follicular center lymphoma, lymphoblastic, Burkitt's, and nodular poorly differentiated lymphocytic lymphomas, and chronic myelogenous leukemias in lymphoid blast crisis [19-22]. CD10 expression has also been reported in several nonhematopoietic neoplasms including renal cell carcinoma, endometrial stromal tumor, solid and pseudopapillary tumor of the pancreas, melanoma, carcinoma of the prostate, breast, stomach, and colon [16,23-32].

CD10 has a neutral endopeptidase activitiy and is known to regulate biological activities of peptide substrates including atrial natriuretic factor, endothelin, oxytocin, bradykinin, substance P angiotensins, enkephalins and bombessin-like peptides by reducing the local concentrations available for receptor binding and signal transduction [9,10,33,34]. Recent evidence also demonstrated a correlation between apoptosis and CD10 expression [35-38]. It may also play an important role in maintenance of homeostasis, neoplastic transformation, and tumor progression [39-41]. CD10 expression in intratumoral stromal cells may also contribute to tumor progression [42].

To date, only a few studies have investigated CD10 expression in urothelial carcinoma of the urinary bladder [43-46]. In the current study, we evaluated 371 urothelial carcinomas of the urinary bladder and sought to determine the correlation, if any, between IHC CD10 expression and histopathologic parameters including grade and stage and to find out whether CD10 expression could have a prognostic value in the assessment of urothelial cancer.

## Methods

371 consecutive cases of papillary urothelial neoplasms of the bladder were included in this study. All tumor specimens were from transurethral resections. HE stained sections from formalin-fixed, paraffin-embedded material were reevaluated histopathologically. Tumors were subclassified as papillary neoplasm of low malignant potential, as low-grade papillary carcinoma, or as high-grade papillary carcinoma according to the WHO 2004 grading system [47]. The TNM system was used for pathologic staging: Ta, noninvasive papillary urothelial carcinoma; T1, tumor invades subepithelial connective tissue; T2, tumor invades muscularis propria, and T3, tumor invades perivesical tissue [48].

IHC was performed using a streptavidin-biotin-peroxidase technique (UltraVision, Lab Vision, Fremont, CA, USA) with a monoclonal antibody to CD10 (NeoMarkers, Lab Vision, Fremont, CA, USA). 5-μm sections from paraffin-embedded samples were cut on poly-L-lysine coated slides, deparaffinized in xylene, and then dehydrated. For antigen retrieval, the slides were treated by microwave heating in citrate buffer (pH 6.0) for 10 minutes. 3% hydrogen peroxide was used for blocking endogenous peroxidase activity. The sections were incubated with primary antibody including CD10 at 1:50 dilution for one hour at room temperature. After washing in phosphate buffered saline, the samples were incubated with a biotin conjugated secondary antibody and then incubated using streptavidin-biotin system for 30 minutes at room temperature. The reactions became visible after immersion of the specimens in diaminobenzidine tetrahydrochloride. The sections were counterstained with hematoxylin, then rinsed and mounted. Sections from nonneoplastic bladder mucosa were included as controls. Additional sections of renal tissue were also used as positive control. Staining of the cell membrane and/or cytoplasm was considered positive expression. A semiquantitative scoring based on the percentage of positive cells was performed according to the following staining criteria: -, negative (<5%); 1+ (5-50%); and 2+ (>50%).

Statistical analysis was performed using Statistical Package for Social Sciences v 10.0 (SPSS Inc., Chicago, IL, USA) software for Windows. Statistical significance of the results was evaluated by χ^2 ^test. P values <0.05 were considered statistically significant.

## Results

The age of the patients ranged from 24 to 88 (mean ± SD 61.36 ± 9.74). Among 371 cases, 330 were (88.9%) male and 41 (11.1%) female with an average male to female ratio of 8:1. All 371 bladder neoplasms were low- or high-grade papillary urothelial carcinomas; no case of papillary neoplasm of low malignant potential was detected. 222 cases displayed low-grade morphology while 149 were high grade carcinomas. Considering the pathologic stages, 279 patients had pTa, 53 pT1, and 39 pT2-3. Only 2 cases were stage pT3 in the pT2-3 group.

Predominant IHC CD10 staining was cytoplasmic, but membranous staining was also observed. Table 1 shows the overall CD10 expression according to the histologic grade and pathologic stage. 157 of the 371 cases (42.3%) showed CD10 immunostaining while 214 cases (57.7%) were negative for CD10. C010 immunoreaction was higher with higher histologic grade (p < 0.0001). CD10 expression was also significantly higher in stages pT1 and pT2-3 than in stage pTa (p < 0.0001).

**Table 1:** Overall CD10 expression according to the histologic grade and pathologic stage

	CD10				Total
		
	Negative No. (%)		Positive No. (%)		
Grade					
Low-grade	186	(83.8%)	36	(16.2%)	222 (59.8%)
High-grade	28	(18.8%)	121*	**(81.2%)**	149 (40.2%)
					
Stage					
pTa	204	(73.1%)	75	(26.9%)	279 (75.2%)
pT1	8	(15.1%)	45*	**(84.9%)**	53 (14.3%)
pT2-3	2	(5.1%)	37*	**(94.9%)**	39 (10.5%)

*High-grade vs. low-grade; pT1 and pT2-3 vs. pTa: p < 0.0001

**Figure 1:**
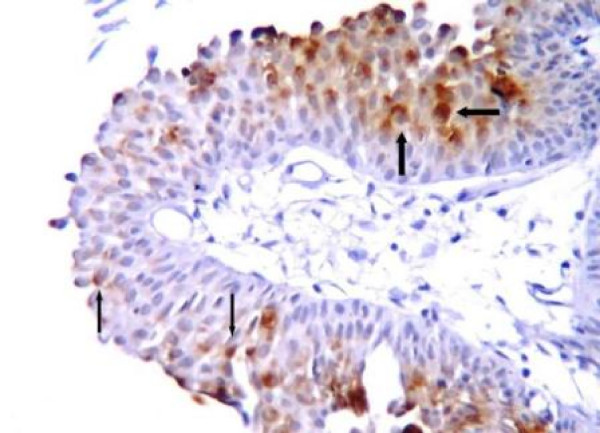
**Noninvasive (pTa) papillary urothelial carcinoma demonstrating strong (*thick arrows*) and weak (*thin arrows*) CD10 immunostaining**.

**Figure 2:**
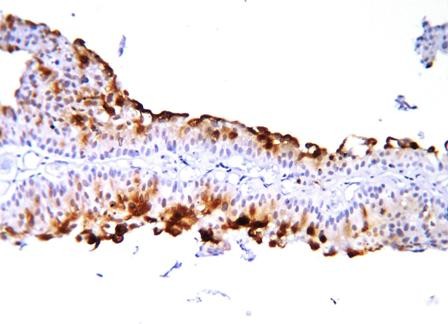
**Another example of noninvasive (pTa) papillary urothelial carcinoma showing strong CD10 expression**.

**Figure 3:**
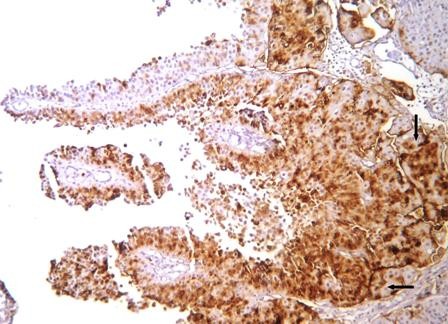
**CD10 expression in invasive (pT1) papillary urothelial carcinoma**. Intense staining corresponds to cells with high grade histology (*arrows*).

**Figure 4:**
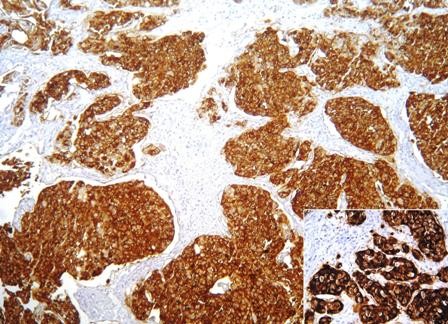
**Diffuse CD10 immunostaining in invasive (pT2) urothelial carcinoma**. Membranous and cytoplasmic staining is best appreciated in the inset.

Table 2 demonstrates the individual CD10 staining scores in 157 cases with positive expression according to the histologic grade and pathologic stage. 1+ expression was seen in 65 cases (41.4%) while 92 cases (58.6%) demonstrated 2+ staining. 2+ immunostaining strongly correlated with high grade (p < 0.0001). pT1 and pT2-3 tumors also significantly correlated with 2+ immunostaining (p < 0.0001) (Figure 1, Figure 2, Figure 3, Figure 4).

**Table 2:** CD10 staining scores in cases with positive expression according to the histologic grade and pathologic stage

	CD10				Total
		
	1+ No. (%)		2+ No. (%)		
Grade					
Low-grade	35	(97.2%)	1	(2.8%)	36 (22.9%)
High-grade	30	(24.8%)	91*	**(75.2%)**	121 (77.1%)
					
Stage					
pTa	45	(60.0%)	30	(40.0%)	75 (47.8%)
pT1	13	(28.9%)	32*	**(71.1%)**	45 (28.7%)
pT2-3	7	(18.9%)	30*	**(81.1%)**	37 (23.5%)

*High-grade vs. low-grade; pT1 and pT2-3 vs. pTa: p < 0.0001

## Discussion

High recurrence rate in urothelial carcinoma of the bladder is a vital problem despite the improving treatment options. The risk of bladder cancer recurrence following initial resection in patients with Ta or T1 tumors may be as high as 80% [7]. A number of molecular markers reported to be of prognostic value have been well-studied. Including these are blood group antigens, tumor suppressor gene p53, cell cycle regulator proteins p27 and cyclin E, E-cadherin, CD44 and many others [49-58]. However, there are still conflicting results although some of them may be attributed to different staining protocols or patient selections [59].

In the current study, we demonstrated CD10 IHC expression in 42.3% of the urothelial carcinomas of the bladder. CD10 staining of the malignant cells revealed a strong correlation not only with histologic grade but also with pathologic stage. Moreover, percentage of CD10 staining appeared to increase with higher grade; 91 of the 121 high grade carcinomas showed 2+ reaction. Vast majority of the low-grade carcinomas had 1+ staining pattern. The same was true for invasive tumors: 2+ reaction was seen in 32 of 45 pT1 carcinomas and in 30 of 37 pT2-3 carcinomas. Although pTa tumors mainly expressed 1+ reaction, a somewhat balanced distribution was observed in staining scores in this group.

The first study assessing CD10 activity in bladder was performed by Koiso et al [43]. They found that both enzyme activity and IHC expression were higher in superficial cancers than invasive cancers and normal urothelium. They concluded that CD10 was expressed at a certain stage of differentiation in the course of neoplastic process. The absence of IHC CD10 expression in nonneoplastic urothelium was shown after the development of CD10 monoclonal antibody appropriate for paraffin-embedded tissues [33]. Chu and Arber reported positive cytoplasmic staining in 13 of 24 (54%) urothelial carcinomas, while there was no reaction in nonneoplastic tissues [23]. However, the correlation of CD10 expression with pathologic stage or histologic grade was not investigated in that study. These results suggest that neoplastic tissues rather than nonneoplastic epithelium have a propensity for CD10 expression. Conversely, Murali et al demonstrated CD10 staining in 5 of the 10 cases of nonneoplastic urothelium [44]. Despite the small number of cases, they found CD10 expression in 80% of invasive carcinomas and also proved that the staining intensity for the high-grade group (including invasive carcinoma, high-grade papillary urothelial carcinoma, and carcinoma in situ) was statistically higher than that of the low-grade group (including low-grade papillary urothelial carcinoma, papillary urothelial neoplasm of low malignant potential and normal urothelium). In another study by Bircan et al, 34 of 79 (43%) urothelial carcinomas and only one case of nonneoplastic epithelium showed CD10 staining [45]. They found an inverse correlation between CD10 expression and tumor stage, but no association with histologic grade or staining score was detected. The authors proposed that the higher level of CD10 expression in noninvasive carcinomas appears to inhibit cell invasion. In a more recent series of 49 cases by Abdou et al, CD10 expression significantly correlated with some parameters including advanced stage, tumor size, and shorter mean survival but not with grade [46]. The authors suggested that CD10 appears to be associated with tumor progression and that it could play a pivotal role in bladder cancer pathogenesis, a proposal also supported by our findings. Apparently, our study-in which CD10 staining increased with grade and stage-bears some similarities with and shows some differences from these previous reports.

There may be several possibilities about the role of CD10 in urothelial tumorigenesis. First, CD10 is a cell surface metalloprotease and one can easily postulate that CD10 expressing tumors have the capacity to create a microenvironment that facilitates cancer cell invasion and metastasis [42,60,61]. This appears the most likely explanation for the significant correlation of CD10 with grade and stage in our study. If this is the case, it may also be speculated that since invasive bladder carcinomas most likely originate from high-grade noninvasive lesions rather from low-grade tumors [62-64], tumors with high grade and stage seem more likely to express CD10. Another possible mechanism is that the increased IHC CD10 expression with increasing grade and stage may indicate accumulation of mutated, nonfunctional CD10 rather than its normal counterpart [44].

On the other hand, it was recently found that CD10 expression was associated with higher apoptotic index in diffuse large B cell lymphoma [65]. Several hypothesis have been proposed about the potential role of CD10 during apoptosis. CD10 could prevent the cells' possible rescue from apoptosis by degrading cytokines capable of delivering anti-apoptotic signals, or by degrading peptide signals [37]. Moreover, CD10 expression may take part in preventing unwanted inflammatory reaction initiated by activated cells undergoing apoptosis, and it may protect these cells from potential attacks by the immune system [37,66]. Consequently, the contribution of CD10 to the neoplastic process appears multifaceted and may be related with other additional factors.

Indeed, the results of several studies in human tumors other than urothelial carcinoma have also revealed that changes in CD10 activity produce different influences in different tumor types. Neoplasms arising from tissues that normally express CD10 such as endometrial stromal tumors, renal cell carcinoma, trophoblastic tumors, and solid and pseudopapillary tumor of the pancreas may also express this antigen [23-27,67,68]. CD10 also acts as a key marker in differentiating endometrial stromal sarcoma from smooth muscle tumors [24]. By contrast, low or absent CD10 expression have been reported in several tumors including small cell and non-small cell carcinoma of the lung, and carcinomas of breast, stomach, and colon [18,42,69-71]. It has been shown that CD10 promotes apoptosis and inhibits cell migration in prostate carcinoma [38,72]. Accordingly, CD10 expression diminishes with tumor progression, particularly in metastatic, androgen-independent prostate cancers [41]. On the other hand, studies in melanomas have shown that CD10 expression correlates with tumor progression and metastasis [29-31]. Bilalovic et al found that CD10 expression was significantly higher in primary melanomas with higher Clark level and larger Breslow thickness [31]. These findings are somewhat parallel to our results, since Clark level and larger Breslow thickness are two important prognostic determinates associated with metastasis in melanoma and the same is true for tumor grade and stage in bladder carcinoma. Stromal CD10 expression has also been observed in colorectal adenomas and carcinomas, and in invasive ductal carcinomas of the breast but not in normal tissues, a finding supporting the hypothesis that CD10 may facilitate invasion and metastasis [70,71,42]. Therefore, it appears that CD10 may contribute to the neoplastic processes in different tissue types, probably by different or incompletely understood mechanisms.

## Conclusion

This study represents the largest series of urothelial carcinoma of the bladder examined for CD10 immunoexpression in relation to histologic grade and pathologic stage. We found that CD10 expression is strongly correlated with tumor grade and stage in urothelial carcinoma of the bladder, and that CD10 may be associated with tumor progression in bladder cancer pathogenesis. Although the exact biologic function of CD10 and mechanisms of action by which it regulates neoplastic process as well as its significance in different types of tissues are not yet clear, it may provide an additional parameter for evaluating bladder tumors. Further studies with large number of cases are needed to confirm our results and to elucidate the role and significance of CD10 in urothelial carcinoma.

## Competing interests

The authors declare that they have no competing interests.

## Authors' contributions

BB conducted the design of the study, performed microscopic evaluation, and drafted the manuscript. KB participated in the design of the study, performed microscopic evaluation, and helped to draft the manuscript. SB participated in the design of the study and immunohistochemical evaluation. ERB and SOO conceived of the study, and participated in its design and coordination and helped to draft the manuscript. All authors read and approved the final manuscript.
